# Coronary pseudoaneurysm with a superficial mass and accompanying Brucella infection

**DOI:** 10.1186/s13019-024-02537-w

**Published:** 2024-02-06

**Authors:** Chuanxiao Mi, Xiangxi Zhang, Liyuan Wang, Yan Yun, Fang Li, Yi Li, Yuxin Liu, Shouji Zhang, Chengwei Zou, Haizhou Zhang, Xiaochun Ma

**Affiliations:** 1 Department of Cardiovascular Surgery, Shandong Provincial Hospital Affiliated to Shandong University, No. 324, Jingwu Road, Jinan, Shandong 250021 China; 2 Department of Cardiovascular Surgery, Shandong Provincial Hospital Affiliated to Shandong First Medical University, Shandong University, Jinan, Shandong 250021 China; 3 Department of Radiology, Qilu Hospital of Shandong University, Jinan, China

## Abstract

**Background and aims:**

To our knowledge, no previously reported clinical data of a coronary artery fistula forming a pseudoaneurysm and presenting as a anterior chest wall lump. We reported a rare case of Coronary pseudoaneurysm with a superficial mass and accompanying Brucella infection. The patient was successfully treated with surgery.

**Materials and methods:**

The patient case data was extracted from hospital records.

**Results:**

A 64-year-old male presented with a history of paroxysmal left-sided chest pain and painful anterior chest wall lump. Coronary computed tomography (CT) angiography revealed the RCA pseudoaneurysm that showed a peripherally calcified soft-tissue mass in the anterior mediastinum and communicated with the chest wall lump through intercostal spaces. The patient underwent the resection of chest lump and RCA pseudoaneurysm under cardiopulmonary bypass, along with a combined antimicrobial therapy. The patient was discharged successfully after the surgery.

**Discussion and conclusion:**

We report this rare case and highlight the possible origin of the anterior mediastinal mass and anterior chest wall lump as a pseudoaneurysm formed by a coronary artery fistula.

**Supplementary Information:**

The online version contains supplementary material available at 10.1186/s13019-024-02537-w.

## Case presentation

**Fig. 1:**
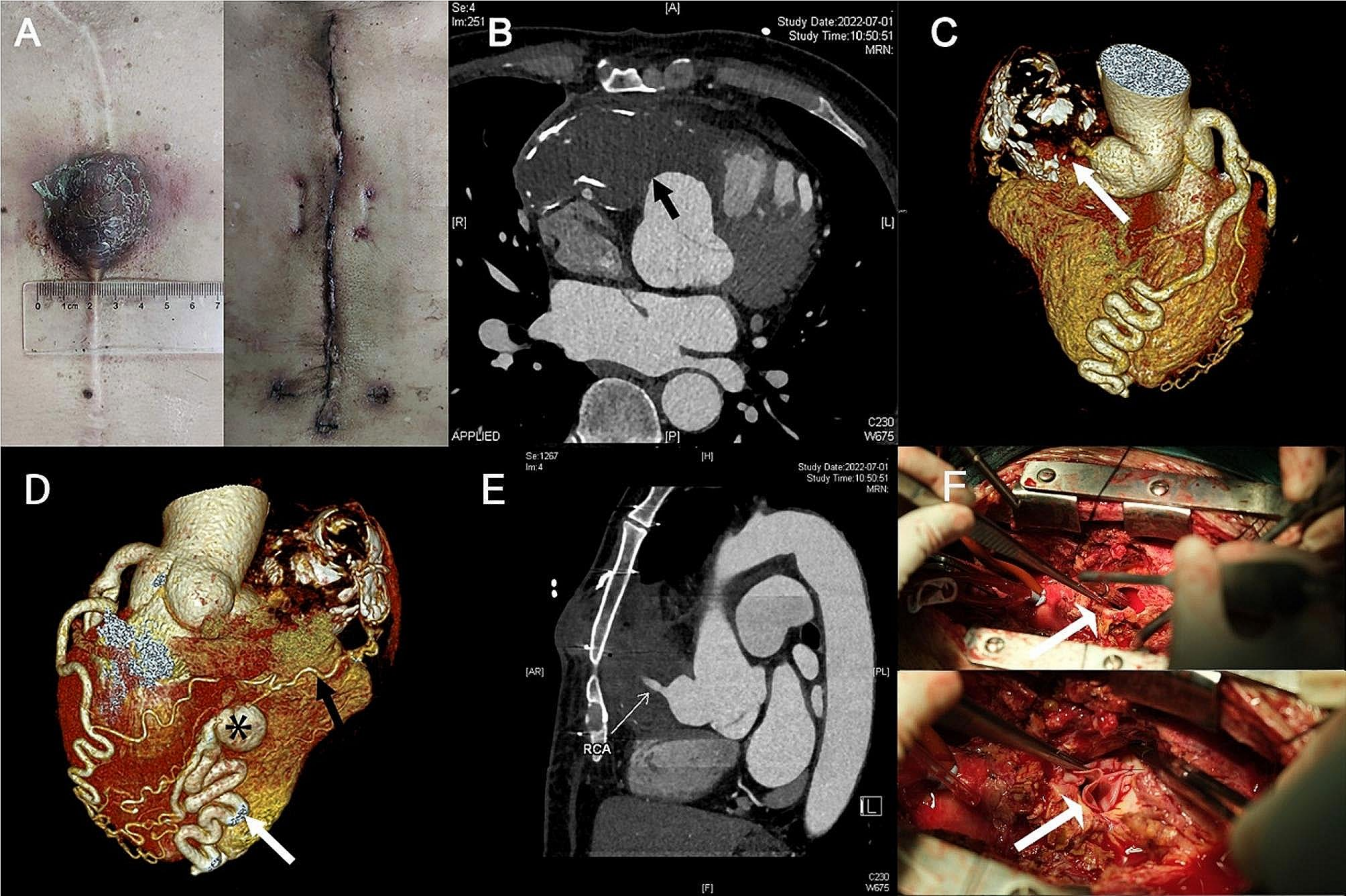
(**A**) A painful lump (4 × 3 × 2.5 cm in size) on the anterior median chest wall was surgically resected. (**B**, **C**) A preoperative computed tomography angiography (CTA) scan for coronary artery and thoracic aorta detecting a peripherally calcified pseudoaneurysm of RCA in the anterior mediastinum (a maximum cross-section area of 9.0 × 4.8 cm) (black and white arrows: pseudoaneurysm). (**D**) The CTA scan showing a left anterior descending artery (LAD)-right ventricular fistula, the tortuous and dilated LAD and a tiny fistula orifice being 0.5 cm in width, the collateral circulation from obtuse marginal branch (OM) to distal RCA (white arrows: LAD; black asterisk: fistula; black arrows: the collateral circulation). (**E**) The CTA confirming the complete occlusion of proximal RCA and no perfusion from RCA to pseudoaneurysm (white arrows). (**F**) The hematoma and caseous necrotic tissue intraoperatively removed from the pseudoaneurysm and the orifice of RCA to pseudoaneurysm patched with a bovine pericardial patch (white arrows)

A 64-year-old male was hospitalized with a chief complaint of paroxysmal left-sided chest pain for 6 months and a painful lump (4 × 3 × 2.5 cm in size) on the anterior median chest wall for half a month (Fig. 1A). He was diagnosed 37 years ago with a right coronary artery (RCA)-right ventricular fistula and underwent the surgical closure of the fistula by ligating the RCA near its entrance to the right ventricle. Firstly after admission, a preoperative computed tomography angiography (CTA) scan for coronary artery and thoracic aorta detected the pseudoaneurysm of RCA that showed a peripherally calcified soft-tissue mass in the anterior mediastinum (maximum cross-section area of 9.0 × 4.8 cm)(Fig. 1B, C). A left anterior descending artery (LAD)-right ventricular fistula was also confirmed by the CTA scan which illustrated the tortuous and dilated LAD and a tiny fistula orifice being 0.5 cm in width (Fig. 1D, Supplementary Fig. 1A). Next, the coronary angiogram (CAG) showed the complete occlusion of proximal RCA (Fig. 1E, Supplemental Video 1), the collateral circulation from obtuse marginal branch (OM) to distal RCA (Supplementary Fig. 1B, Supplemental Video 2), and no flow from RCA to pseudoaneurysm (Supplementary Fig. 1C, Supplemental Video 1). Additionally, the preoperative echocardiography displayed normal cardiac chamber diameters and function.

Subsequently, a disposable sterile syringe was used to puncture into the lump for extracting the fluid from it. Approximately 2 ml extracted fluid was then sent to the laboratory for examination. The cultures of fluid from chest lump were positive for Brucella abortus and the antibodies was also detected positive in plasma with titers > 1:100. The blood cultures were negative and the patient reported no history of fever. Therefore, a combined antimicrobial treatment with doxycycline, rifampicin, amikacin and sulfamethoxazole was administrated for the patient six weeks before surgery.

The patient underwent the resection of chest lump and pseudoaneurysm of RCA under cardiopulmonary bypass and general anesthesia. After the right femoral artery and vein were dissected, arterial and venous cannulas were then inserted and cardiopulmonary bypass was established. A longitudinal incision was made along the midline of the sternum (in accordance with the original incision of previous surgery). The chest lump communicated with the pseudoaneurysm through the left 4th and 5th intercostal spaces. The sternum was then split using a saw. After exposing the pseudoaneurysm, the ascending aorta was dissected and clamped. Del Nido cardioplegic solution was used in antegrade perfusion for myocardial protection. The hematoma and caseous necrotic tissue were removed from the pseudoaneurysm. The orifice of RCA was patched with a bovine pericardial patch and the entrance to the pseudoaneurysm was closed (Fig. 1F). The duration for cardiopulmonary bypass and aorta clamping was 125 min and 29 min. The postoperative pathological examination confirmed that the excised tissue was hematoma and the wall of pseudoaneurysm. The patient experienced an uneventful recovery before discharge from hospital.

## Discussion

Coronary artery fistula (CAF) is an anomalous connection between coronary arteries and either heart chambers or great vessels [1]. It is a rare cardiac malformation and was first described by Krause in 1865 [2]. The etiology of CAF can be categorized into congenital (more than 90%) and acquired. Congenital CAFs are present at birth and are usually diagnosed in childhood or early adulthood. Acquired CAFs can result from trauma, infections, or as a complication of invasive cardiac procedures. About a half of patients with CAF might have clinical symptoms such as chest pain, dyspnea, debility, and limitations in daily activities, while the rest might report no specific manifestations [3]. The complications of CAF include angina pectoris, myocardial infarction, coronary artery aneurysm/pseudoaneurysm, bacterial endocarditis and congestive heart failure, and etc. [4]. Echocardiography can be helpful in establishing a primary diagnosis of CAF, coronary computed tomography angiography and cardiac magnetic resonance imaging can depict the fistula better, and the golden standard for diagnoses of CAF is still coronary catheterization and coronary angiography [5]. 

In adults with coronary artery fistulas, the American Heart Association describes in its guidelines for the management of adults with congenital heart disease (2008) that large coronary artery fistulas should be treated surgically regardless of whether they are symptomatic. Small fistulas should also be treated surgically if the patient has associated arrhythmias, cardiac insufficiency, myocardial ischaemia, or if they are complicated by infective endocarditis [6]. Providing different patients conditions, CAF can be closed by two common surgical procedures, transcatheter coronary artery fistula closure and open coronary artery fistula ligation [7]. 

Here we described an elderly male patient who presented again with a giant RCA pseudoaneurysm 37 years after surgical treatment of a right coronary artery (RCA)-right ventricular fistula. And a painful lump on the anterior median chest wall was formed that communicated with the RCA pseudoaneurysm through the left 4th and 5th intercostal spaces. In addition to the impressive size and images of the lesion, this patient also had accompanying bacteria Brucella infection, making this case even more uncommon. To our knowledge, this has not been reported in the literature before regarding CAFs combined with pseudoaneurysms and bacterial infection and the formation of a chest mass on the surface of the body. In this case, we performed the surgical resection of chest lump and pseudoaneurysm of RCA. Intraoperative exploration revealed the severe calcification of the aneurysmal wall, hematoma and inflammatory tissue inside the aneurysm. This might be related to the patient’s infection with bacteria Brucella.

It is difficult to ascertain the detailed circumstances when the surgery was operated 37 years ago. Due to technological limitations at that time (extracorporeal circulation, myocardial protection, coronary angiography, echocardiography, and CTA) and limited surgical experience, it is speculated that the ligation for RCA-RV fistula might not have been complete 37 years ago. This is why patch repair is now considered more reliable than ligation. Additionally, also due to the limitations in diagnostic technology and experience at that time, it is possible that an LAD-RV fistula was not discovered and therefore not treated. In this surgery, the LAD-RV fistula was not surgically corrected. Firstly, the fistula was small and its impact on hemodynamics was minimal. Preoperative cardiac echocardiography confirmed that the heart chamber size and function were basically normal. Secondly, this was a re-do cardiac surgery, and the patient had a large pseudoaneurysm and severe pericardial adhesion, making it extremely difficult to address the LAD-RV fistula.

Currently, we do not know how this patient was infected with Brucella abortus. The source and route of infection are still unclear. After carefully inquiring about the patient’s medical history and contact history, we speculate that the infection might have originated from livestock, as the patient is a farmer and has previously raised sheep at home. Brucella has not been detected in the patient’s blood but the infection might accelerate the dilation and rupture of the coronary artery wall, leading to the formation of a pseudoaneurysm.

Due to its rarity and nonspecific signs, it is worth noting that giant coronary artery pseudoaneurysm originating from coronary artery fistula could manifest as anterior mediastinal mass and anterior chest wall lump, which warrants surgical intervention. Besides, coronary artery pseudoaneurysm might be complicated by bacterial infection and should be treated with antiinfectious drugs [8, 9]. Even in China, infections caused by Brucella bacteria are rare. We consulted with an infectious disease specialist from the hospital and prescribed antimicrobial drugs for this patient before surgery. All four of the antibiotics mentioned (including doxycycline, rifampicin, amikacin and sulfamethoxazole) specifically targeted against Brucella bacteria and were administered in sufficient doses and treatment duration. Because the patient underwent heart surgery and had implants (bovine pericardial patch) in the heart, we were concerned about the possibility of postoperative infection spreading and causing bacteremia, sepsis, or even septic shock. We were also worried about the potential for implant-related infections or severe infective endocarditis in this patient. Fortunately, after receiving our antimicrobial treatment, the postoperative infection was fully controlled.

### Electronic supplementary material

Below is the link to the electronic supplementary material.

Supplemental Video 1: The coronary angiogram (CAG) showing the complete occlusion of proximal RCA and no flow from RCA to pseudoaneurysm

Supplemental Video 2: The coronary angiogram (CAG) confirming the collateral circulation from obtuse marginal branch (OM) to distal RCA

Supplementary Figure 1: (**A**) The CTA scan showing the tortuous and dilated left anterior descending artery (LAD) (white arrows: LAD). (**B**) The coronary angiogram (CAG) depicting the collateral circulation from obtuse marginal branch (OM) to distal RCA (white arrows). (**C**) The CAG confirming the complete occlusion of proximal RCA and no perfusion from RCA to pseudoaneurysm (black arrows)

## Data Availability

The patient case data was extracted from hospital records. All data included in this research are available upon request by contact with the corresponding author.
