# Supplementary figures and images for: Coronary pseudoaneurysm with a superficial mass and accompanying Brucella infection
Source: J Cardiothorac Surg. 2024 Feb 6;19:63. doi: 10.1186/s13019-024-02537-w (PMC10845731; doi:10.1186/s13019-024-02537-w)

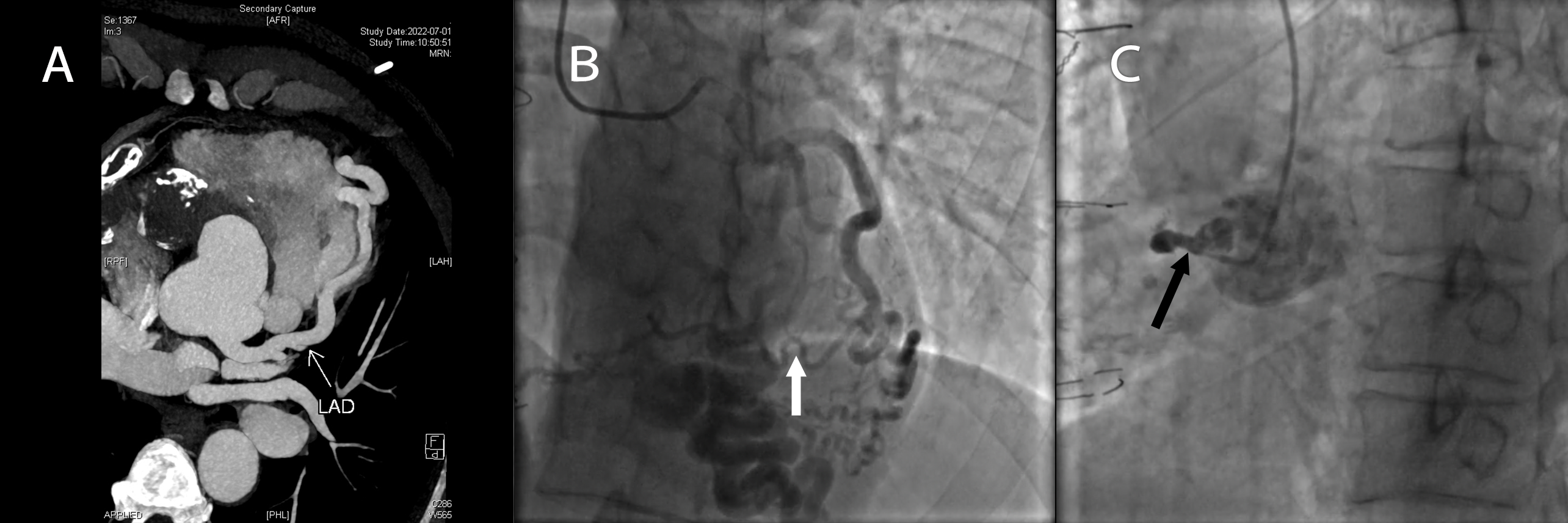

Supplement: Supplementary file 3 — Supplementary Figure 1: (A) The CTA scan showing the tortuous and dilated left anterior descending artery (LAD) (white arrows: LAD). (B) The coronary angiogram (CAG) depicting the collateral circulation from obtuse marginal branch (OM) to distal RCA (white arrows). (C) The CAG confirming the complete occlusion of proximal RCA and no perfusion from RCA to pseudoaneurysm (black arrows) [file 13019_2024_2537_MOESM3_ESM.png]
